# The Concours

**Published:** 1850-01

**Authors:** 


### ARTICLE VI.

### THE CONCOURS.

It will be observed by the foregoing notice that the recom mendation of the National Medical Association so unanimously adopted in reference to the appointment of Teachers is to be complied with by one medical School at least. Thus, Rush Medical College will be first in the United States, to hold a public concours or trial, for the selection of a Medical teacher, but we opine she will not be the last.

Among the important recommendations of the National Medical Association, none it seems to us will be more likely to control the rapid multiplication of Medical Schools, and improve the character of the teachers in those that are permanently organized, than this one, if it should be generally adopted. It will accomplish these ends by affording every one who has an ambition to become a teacher, an opportunity to compete for a place in a well organized school, and few men of ability would be found contending with the perplexities of getting up a new school, if such a plain road to preferment were open before them. And it will secure a great improvement upon the appointments that are often made, by enabling those who select to know something of the ability of the one selected to discharge the duties of his station. E.

